# The complete chloroplast genome of *Epimedium fargesii* Franch. (Berberidaceae), a rare plant species endemic to China

**DOI:** 10.1080/23802359.2021.1973922

**Published:** 2021-10-27

**Authors:** Xiang Liu, Yixin Zhang, Cheng Zhang, Chaoqun Xu, Weihan Qin, Guoan Shen, Baolin Guo

**Affiliations:** a Institute of Medicinal Plant Development, Chinese Academy of Medical Science, Peking Union Medical College, Beijing, China; b Chongqing Academy of Chinese Materia Medica, Chongqing, China

**Keywords:** Chloroplast genome, *Epimedium fargesii*, infrageneric classification, phylogenetic analysis, Berberidaceae

## Abstract

*Epimedium* L. is a medicinally important herbaceous genus in the family Berberidaceae. *Epimedium fargesii* Franch. is only narrowly inhabited in the Daba Mountains in China. Here, we sequenced and assembled the first complete chloroplast genome of *Epimedium fargesii* Franch. The chloroplast genome of *E. fargesii* was 157,208 bp in length, with a total GC content of 38.77%. A total of 112 unique genes were identified, including 78 protein-coding genes, 30 tRNA genes, and four rRNA genes. Phylogenetic analysis indicated that *E. fargesii* formed a sister relationship with *E. wushanense* T. S. Ying. Our results provided fundamental data for further taxonomic and phylogenetic research of the genus *Epimedium*.

*Epimedium* L. is the largest herbaceous genus of family Berberidaceae, with more than 60 plant species distributed unevenly from North Africa (Algeria) to East Asia (Stearn 2002; Ying 2002). However, there is a long history of controversies over the infrageneric classification of *Epimedium* genus since Linnaeus first identified the *Epimedium* species, *E. alpinum*, in 1753 (Linnaeus 1753). The genus was divided into two subgenera (subgenus *Rhizophyllum* and subgenus *Epimedium*), four sections (section *Diphyllum*, section *Macroceras*, section *Polyphyllum*, and section *Epimedium*) based on the C-banding of chromosomes, flower and leaf morphology, and geographical distribution in the updated classification system proposed by Stearn (2002). Furthermore, section *Diphyllum* (the largest section which is comprised completely of more than 50 Chinese *Epimedium* species) was subdivided into four series based on floral morphology (Stearn 2002; De Smet et al. 2012). *Epimedium* plants are used as important medicinal plants in the traditional Chinese medicine, because their leaves, known as ‘Herba Epimedii’, have special nourishing effect on kidney, bones, and muscles. In recent years, their activities of anti-osteoporosis, anti-tumor, and improving immunological function have been verified with modern pharmacological studies (Cai et al. 2011; Ma et al. 2011; Tong et al. 2011; Jiang et al. 2015; Yang et al. 2019).

The taxonomic classification of genus *Epimedium* still remain an intractable status since the interspecific hybridization and gene introgression has greatly complicated the interspecific phylogenetic relationship. Chloroplast genomes are important tool used in phylogenetic analysis for their special advantages such as small size, mostly single-copied, moderate nucleotide substitution rate, and high conservation in terms of gene composition, genome structure, and collinearity (Zhang and Li 2011). Therefore, to characterize more chloroplast genomes of *Epimedium* species is critical for dissecting the phylogenetic relationship within *Epimedium* genus.

*Epimedium fargesii* Franch. is a species only distributed in the central part of the Daba Mountains (mainly in the Wuxi County, Chengkou County, and Kai County of Chongqing city, and the Wanyuan County of Sichuan province, China) and it is used as ‘Herba Epimedii’ by local people. In 1884, Franchet (French botanist) published *E. fargesii* based on the type sample collected by Paul Farges (a French missionary) (Franchet 1886). *Epimedium fargesii* is unique among *Epimedium* species on account of its reflexed, narrow lanceolate inner sepals, and stamens 8–10 mm long with filaments longer than anthers (this kind of stamen only so far occurs in *E. fargesii*, *E. dolichostemon*, and *E. dewuense* within Epimedium genus). In the current study, we reported the first chloroplast genome of *E. fargesii* in order to provide more useful data for phylogenetic and taxonomic research in the genus *Epimedium*.

For this study, the *E. fargesii* was collected from the Wuxi County of Chongqing city, China (latitude 31.5685 and longitude 108.9822). A specimen and the extracted DNA were deposited at Medicinal Plants Authentication Center, Institute of Medicinal Plant Development, Chinese Academy of Medical Science, Beijing, China (http://www.implad.ac.cn/, collected by Baolin Guo, blguo@implad.ac.cn) under the voucher number B. L. Guo 0448. Total genomic DNA was extracted from the fresh leaves of *E. fargesii* with the modified CTAB method (Doyle and Doyle 1987), and was then sheared to an average size of 300 bp for library construction using the VAHTSTM Universal DNA Library Pren Kit (ExCell Bio. Biological Technology Co., Ltd., Shanghai, China). Genome sequencing was conducted through the Illumina Novaseq 6000 platform (Illumina Inc., San Diego, CA), and 150 bp paired-end reads were generated. The assembly of chloroplast genome was performed on the GetOrganelle v1.5 program (Jin et al. 2018) with *E. acuminatum* (GenBank accession number: NC_029941) as reference. The annotation of chloroplast genome was performed through the online program CPGAVAS2 (Shi et al. 2019) and assisted with manual correction. The annotated genomic sequence was deposited into GenBank with an accession number MW483086.

The complete chloroplast genome of *E. fargesii* was 157,208 bp in length, consisting of a large single copy region (LSC, 88,543 bp), a small single copy region (SSC, 17,079 bp), and two inverted repeat regions (IR_A_ and IR_B_, 25,793 bp). The total GC content was 38.77%, with IR regions (43.17%) higher than that in LSC (37.37%) and SSC regions (32.73%). A total of 112 unique genes were identified from the chloroplast genome of *E. fargesii*, including 78 protein-coding genes, 30 tRNA genes, and four rRNA genes. The intron–exon structure analysis indicated that a total of 18 genes have introns, among which 15 genes (*pet*B, *pet*D, *rpl*16, *rp*l2, *rpo*C1, *rps*16, *trn*A-UGC, *trn*G-UCC, *trn*I-GAU, *trn*K-UUU, *trn*L-UAA, *trn*V-UAC, *atp*F, *ndh*A, and *ndh*B) contain one intron, whereas *ycf*3, *rps*12, and *clp*P contain two introns.

For identification of phylogenetic position of *E. fargesii*, phylogenetic analysis was carried out using the complete chloroplast genome sequences of *E. fargesii* and other 10 species from the NCBI GenBank database. MAFFT v7 (Katoh et al. 2019) was applied for sequence alignment and then a maximum-likelihood tree was constructed by using IQ-TREE multicore v 2.0.3 (Minh et al. 2020) with *Vancouveria planipetala* Calloni as the outgroup species (Figure 1). The Phylogenetic tree demonstrated that *E. fargesii* formed a sister relationship with *E. wushanense* T. S. Ying. Our study provided essential data for future phylogenetic studies of *Epimedium* genus.

**Figure 1.**
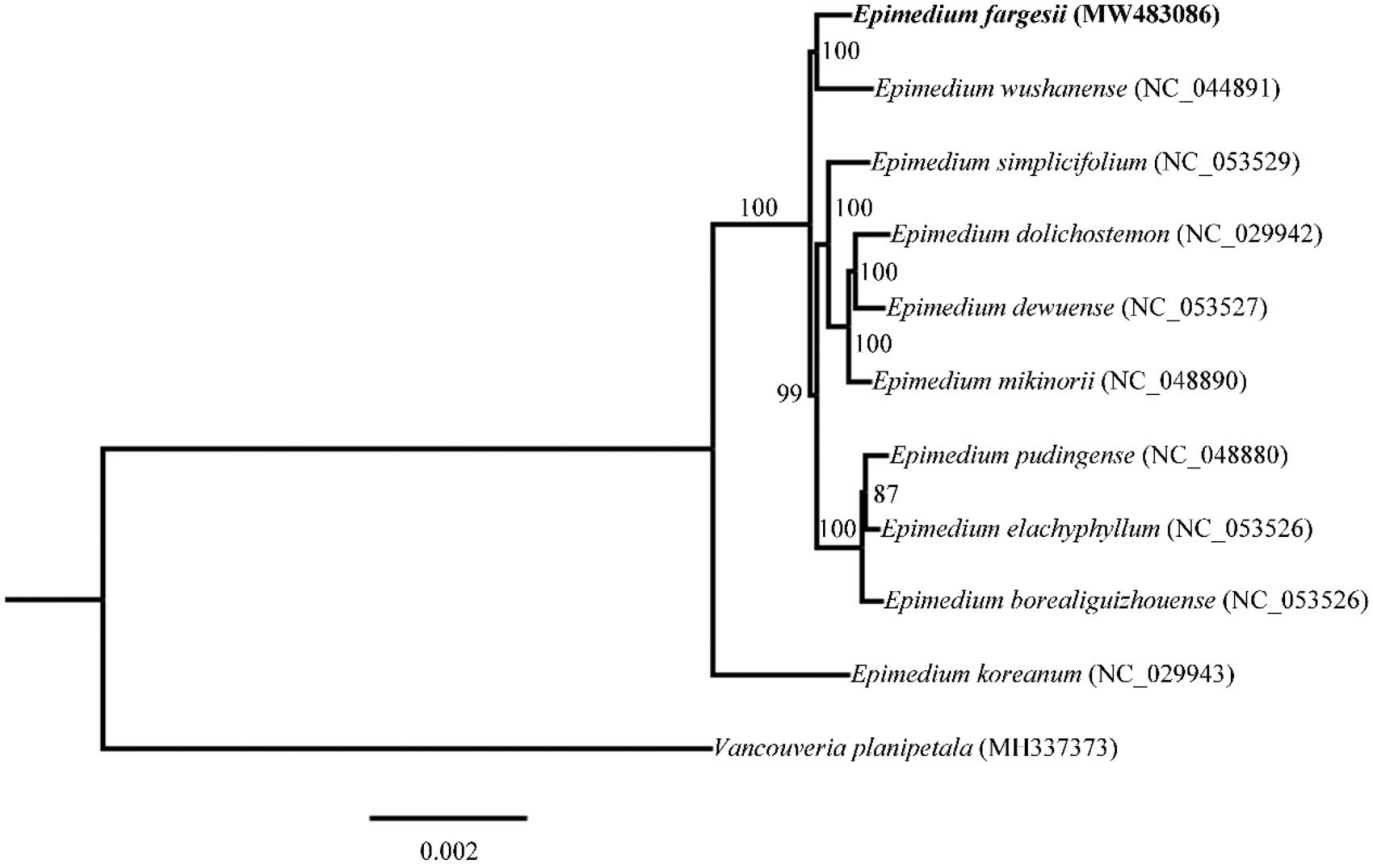
Maximum-likelihood (ML) phylogenetic tree based on complete chloroplast genomes of 11 species, with *Vancouveria planipetala* as outgroup. Numbers at nodes represent bootstrap values.

## Data Availability

The genome sequence data that support the findings of this study are openly available in GenBank of NCBI at https://www.ncbi.nlm.nih.gov/ under the accession no. MW483086. The associated numbers are PRJNA749733, SRR15254503, and SAMN20399110, respectively.
